# Multi-Level Integration of Environmentally Perturbed Internal Phenotypes Reveals Key Points of Connectivity between Them

**DOI:** 10.3389/fphys.2017.00388

**Published:** 2017-06-12

**Authors:** Nirupama Benis, Soumya K. Kar, Vitor A. P. Martins dos Santos, Mari A. Smits, Dirkjan Schokker, Maria Suarez-Diez

**Affiliations:** ^1^Host Microbe Interactomics, Wageningen University & ResearchWageningen, Netherlands; ^2^Systems and Synthetic Biology, Department of Agrotechnology and Food Sciences, Wageningen University & ResearchWageningen, Netherlands; ^3^Lifeglimmer GmbHBerlin, Germany; ^4^Wageningen Livestock Research, Wageningen University & ResearchWageningen, Netherlands; ^5^Wageningen Bioveterinary Research, Wageningen University & ResearchWageningen, Netherlands

**Keywords:** data integration, internal phenotype, transcriptomics, proteomics, metabolomics, microbiota, gastrointestinal tract, systems biology

## Abstract

The genotype and external phenotype of organisms are linked by so-called internal phenotypes which are influenced by environmental conditions. In this study, we used five existing -omics datasets representing five different layers of internal phenotypes, which were simultaneously measured in dietarily perturbed mice. We performed 10 pair-wise correlation analyses verified with a null model built from randomized data. Subsequently, the inferred networks were merged and literature mined for co-occurrences of identified linked nodes. Densely connected internal phenotypes emerged. Forty-five nodes have links with all other data-types and we denote them “connectivity hubs.” In literature, we found proof of 6% of the 577 connections, suggesting a biological meaning for the observed correlations. The observed connectivities between metabolite and cytokines hubs showed higher numbers of literature hits as compared to the number of literature hits on the connectivities between the microbiota and gene expression internal phenotypes. We conclude that multi-level integrated networks may help to generate hypotheses and to design experiments aiming to further close the gap between genotype and phenotype. We describe and/or hypothesize on the biological relevance of four identified multi-level connectivity hubs.

## Introduction

**Figure 1:**
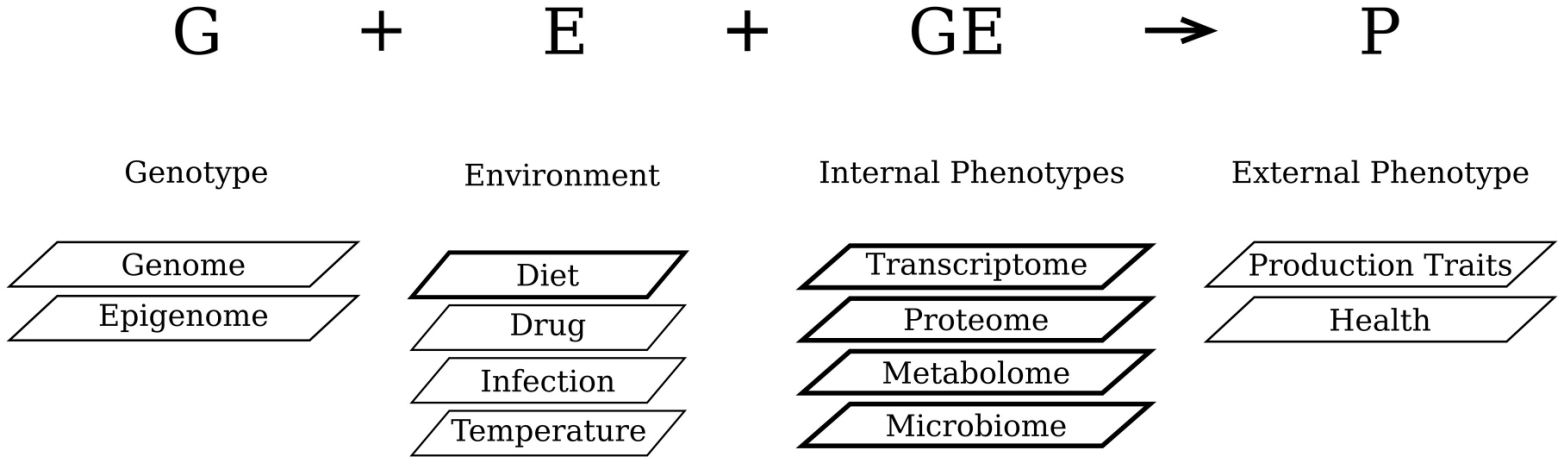
Relationship between the external phenotype (P), the genotype (G), the environment (E), and the G&E interactions. The internal phenotypic layers and the environmental factor with a darker outline are included in the present study.

The information encoded in the genome (genotype) and the external quantitative traits or characteristics (phenotype) of an organism are linked to each other by several layers of so-called, intermediate (Leuchter et al., 2014; Fontanesi, 2016) or internal (Houle et al., 2010) phenotypes. Several of these internal phenotypic layers are shown in Figure 1 that visualizes the conceptual relationship between the external phenotype (P), the genotype (G), the environment (E), and the G&E interactions. The epigenome is tightly associated with the genome and represents the programming of gene expression which is not dependent on the DNA code itself. The transcriptome layer represents direct effects of the environment on the gene expression of the (epi-)genome. Translation of the transcriptome into proteins represents the next internal phenotype. The subsequent layer is represented by complex metabolite profiles. The organism-associated microbiota, especially those in the gut, can be regarded as a separate internal phenotypic layer, because it is not only dependent on the host genome but also heavily influenced by its environment, particularly by nutrition (Schwartz et al., 2012; Montiel-Castro et al., 2013). Although, for several traits the quantitative effects of the environment on the external phenotypes are known (Gentry et al., 2004; Cani et al., 2008; de Wit et al., 2011), the specific effects of the environment on the internal phenotypes are largely unknown. Furthermore, it is obvious to assume that the various layers of internal phenotypes are connected to each other and that their joint profiles ultimately determine the external phenotype (Leuchter et al., 2014; Fontanesi, 2016). Unfortunately, most of these assumptions are not based on solid evidence and at best represent oversimplifications of the dynamic nature of processes involved in determining external phenotypes. It, furthermore, partly explains the knowledge gap that exists between the genotype and the external phenotype.

Therefore, the objective of this study was to develop methodologies to identify components in the internal phenotypic layers that are connected to components in other internal phenotypic layers. To this end, we integrated multi-scale quantitative (–omics) data using a regression approach. The used data sets were derived from a single experiment with inbred mice which were exposed to five different dietary interventions as a means to perturb the different internal phenotypes. With a data-driven approach we were able to identify a large number of potential connections between the various intermediate phenotypes and for several we found proof of causal relationships in literature. We have used networks to represent the identified connections. The molecular components of each internal phenotype (such as genes, metabolites, cytokines, or bacterial groups) are represented as nodes in the network and the identified connections between each data type are represented as links or edges. The results of this study provide a basis to understand how various internal phenotypic layers are connected to each other. The identified connections may be crucial for the identification of causal relationships (Civelek and Lusis, 2014) between various biological scales and to uncover mechanisms involved in determining external phenotypes.

## Materials and methods

### Origin of data

We used data from an experiment with 6-week old inbred mice that were fed for 4 weeks with six different semi-synthetic diets (Kar et al., submitted). In brief: thirty-six 21-day-old C57BL/6J mice (Harlan Laboratories, Horst, the Netherlands) were divided into 6 groups and housed in pairs with *ad libitum* access to diet and water. After adaptation for 1 week to a standard diet, the mice were fed semi-synthetic diets containing 300 g/kg (as fed basis) of one of the alternative protein sources for 28 days: soybean meal; casein; partially delactosed whey powder; spray dried plasma protein; wheat gluten meal and yellow meal worm. At the end of the experiment, mice were sacrificed to collect ileal tissue to acquire gene expression data, ileal digesta to study changes in microbiota, blood serum to profile cytokines and chemokines and blood and urine to profile amine metabolites. All procedures were approved by the Animal Experimentation Board at Wageningen University & Research Center (accession number 2012062.c) and carried out according to the guidelines of the European Council Directive 86/609/EEC dated November, 1986. Multi-omics data were obtained with regards to: whole genome gene expression profiles of ileal tissue as measured with Affymetrix GeneChip mouse gene 1.1 ST microarrays (Affymetrix, Santa Clara, CA, USA); community scale microbiota composition of ileal digesta by targeted-amplicon DNA sequencing of the bacterial 16S rDNA V3 region on an Illumina Mi-Seq sequencer; 23 serum cytokine and chemokine concentrations (pg/ml) using a Bio-Rad Mouse 23-plex kit (Bio-Rad, Hercules, CA, USA); and amine metabolic profiles of serum and urine using an ACQUITY UPLC system coupled online with a Xevo Tandem quadrupole mass spectrometer (Waters) operated using QuanLynx data acquisition software (version 4.1; Waters; Kar et al., in preparation). The data from the ileum reflects the local effects of the dietary interventions, the other three data assess the systemic effects.

### Pre-processing and selection of data

An overview of the five types of data and their specifics are given in Table 1. Supplementary Figure 1 has an overview of all the analytical methods used in this study. Each dataset was pre-processed in a similar way using the R package limma (Smyth, 2005) to find the differentially significant data-points. The data is first log transformed and then this data is fitted to a linear model using the function lmFit (Phipson et al., 2016) which will give back information on the differences between the data-points in different samples and subsequently different comparisons of control vs. treatment. Then we used the function eBayes (Phipson et al., 2016) which applies an empirical Bayes method to compute *p*-values for a t-statistic under the assumption that only 1% of the data-points are differentially regulated among all the data-points in the samples. This *p*-value is then subjected to a Benjamini–Hochberg (Benjamini and Hochberg, 1995) multiple testing correction, also known as a False Discovery Rate (FDR).

**Table 1:** Pre-processing and specificities of each data-type.

**Properties**	**Transcriptomics**	**Microbiota**	**Cytokine**	**Metabolomics serum**	**Metabolomics urine**
Sampling	Ileum	Ileum	Serum	Serum	Urine
Before pre-processing	16,410 ^*^ 33	148 ^*^ 33	23 ^*^ 36	41 ^*^ 36	16 ^*^ 28
After pre-processing	52 ^*^ 33	22 ^*^ 33	13 ^*^ 36	26 ^*^ 36	16 ^*^ 28

*Details of the site of sampling and data dimensions before and after pre-processing are indicated. The first number indicates the number of variables in the data and the second number denotes the number of samples*.

This analysis was done by comparing the data of each dietary group against the data of the dietary group that received soy bean meal as protein source, which is the most common source of protein in animal diets. The FDR value of the data, is used to gauge significance and data-points that were significant in at least one of the five comparisons of the diets were included in the integration analysis. Except for the Cytokine and Metabolomics Serum (using the amine measurement), all the data-types had some samples thrown out due to quality control. Two types of metabolomics measurements were done on the sampled urine; Amine and Acyl-carnitine, the amine dataset did not have sufficient statistically significant data-points so was discarded. We only work with the Acyl-carnitine measurement in urine.

### Data integration, network generation, and network assessment

All significantly different data-points were used in the integration which was initially performed with two datasets at a time, so that from the 5 datasets 10 integrated networks were generated. The integration was performed using the function sPLS (sparse Partial Least Squares) in regression mode with ncomp = 5, from the R package mixOmics (Lê Cao et al., 2009; Dejean et al., 2011; González et al., 2012). The regression mode is used to model causal relationship between variables in both datasets by identifying combinations of variables between both datasets. Weight vectors used in the regression modeling are termed loading vectors. sPLS is used to perform simultaneous variable selection in the two datasets to be integrated and employs LASSO (Least Absolute Shrinkage and Selection Operator) penalization (Tibshirani, 2011) on the loading vectors. This approach requires one data set, ***X*** with *n*_*x*_ elements, to be designated the predictor and the other, ***Y*** with *n*_*y*_ elements, the response. As an output, the approach produces a matrix *Ma*(**X**,***Y***) of size *n*_*x*_ × *n*_*y*_ representing the relevant correlations between both datasets, so that:

(1) $$ma_{ij}\;=\;\{\begin{array}{c} 0,\;if\;Y_{j}\;independent\;of\;X_{i}\\ cor\left(X_{i},\;Y_{j}\right),\;if\;Y_{j}\;dependent\;on\;X_{i} \end{array}\;,\;with\;i\;\in \left\{1,\;\ldots ,\;n_{x}\right\}\;and\;j\;\in \{1,\;\ldots ,\;n_{y}\}$$

Where *cor*(*X*_*i*_, *Y*_*j*_) is Pearson's correlation between elements *i* and *j* from datasets ***X*** and ***Y***, respectively. The correlation is computed across all available samples (here corresponding to dietary exposures).

Since it is not trivial to determine the predictor and response with biological data, we swapped the two types of data to compute *Mb*(***Y***,***X***), a matrix of size *n*_*y*_ × *n*_*x*_ where the roles of ***X*** and ***Y*** have been interchanged. Both matrices, *Ma* and *Mb* where combined into a final matrix *M(**X***,***Y***) size *n*_*x*_ × *n*_*y*_ using

(2) M(X,Y) = Ma(X,Y) + t(Mb(Y,X))

where *t* represents matrix transposition. Thus, non-null elements of the matrix *M(**X***,***Y**)* represent correlations between data types that have been deemed associated. This matrix can be seen as a weighted adjacency matrix representing a network where two nodes *X*_*i*_ and *Y*_*j*_ are connected via an edge if a non-null weight can be assigned to the edge. This weight is represented by the matrix value *m*_*ij*_.

To further prune the network of (possibly) spurious interaction two additional thresholds *(th*_*l*_ < *0*; and *th*_*h*_ > 0) were imposed to obtain an unweighted adjacency matrix *A(**X***, ***Y**)* of size *n*_*x*_ × *n*_*y*_

(3) $$A_{ij}=\{\begin{array}{c} 1\;if\;m_{ij}\geq th_{h}\;or\;m_{ij}\;\leq \;th_{l}\\ 0\;if\;\left|m_{ij}\right|<\left|th_{l}\right|\;and\;m_{ij}\;<\;th_{h} \end{array}$$

were |x| represents the absolute value. *th*_*l*_ and *th*_*h*_ where selected for each network so that only top 5% of the highest (positive) and lowest (negative) weights were kept for building the networks.

Networks represented by these adjacency matrix were transformed into the edge-list format, a two column table of the connected nodes in a network were each row represents an edge and visualized in Cytoscape (Shannon et al., 2003; Ono et al., 2015).

For each pair of integrated datasets a null model of the association networks was constructed using a strategy based on random permutations of measured values (Saccenti et al., 2015). Measured data-points were randomly permuted over samples before data integration to obtain randomized datasets that still retained the same value distribution for each variable. The randomized datasets were then used for data integration following the afore mentioned approach thereby generating randomized associations networks. The process was iterated N_*it*_ = 1,000 times for each pair of datasets; For each iteration, *k*, the values of the dynamic cut-offs (*th*_*lk*_ and *th*_*hk*_) (5% of the highest and lowest correlation) were recorded. For the 10 pairwise combinations of datasets, the values obtained for *th*_*l*_ and *th*_*h*_ obtained using the unpermuted dataset, were compared with the distribution of values of *th*_*lk*_ and *th*_*hk*_ with k = {1,…,N_*it*_} to get networks from the random data to compare to the networks from the biological data.

### Network merging and topological analysis

The 10 networks arising from pair-wise data integration of the 5 data sets were merged in a combined network including all the nodes and edges of the 10 networks. This network is then restricted by only including nodes present in at least two of the separate networks. We used the igraph R package (Csardi and Nepusz, 2006) to further analyze the network, which was treated as non-directed, since no particular directionality was assigned to the edges. We obtained values for the following topological properties of the merged network (Barabasi and Oltvai, 2004; Csardi and Nepusz, 2006; Zhu et al., 2007): Degree: number of neighbors of a given node, that is the number of nodes connected to it. Clustering coefficient of a node is the ratio of the number of connections between the neighbors of a node and the total number of possible connections between said neighbors. Characteristic path length: median of the average distance between a node and all the rest. Network density: ratio between the total number of existing edges and the total number of possible edges (given the number of nodes in the network). Connected components maximal subgraphs in a network such that each node is connected to all the rest by means of network paths. For node level metrics, such as degree or clustering coefficient average values were computed over all nodes. Cytoscape was used for network visualization.

### Literature mining

To investigate the co-occurrence of the names of the connected nodes in the association network, we used the R package rentrez (Winter, 2016). This package searches for selected keywords in PubMed abstracts while making use of the MeSH (Medical Subject Headings) thesaurus to maximize results via the API from NCBI. The search was not restricted to a specific tissue type or organism. These results were examined, although not exhaustively, to find literature evidence of established relationships between nodes connected through identified edges; these were then considered as true positive search results.

The script used to generate all these results will be made available on request. All the above mentioned operations were performed using existing functions from R packages. The different steps involved are represented in Supplementary Figure 1.

## Results

### Analysis of the individual datasets

A dietary intervention was performed on mice where the protein content was changed and multi-omics data were obtained with regard to: whole genome gene expression profiles of ileal tissue (Transcriptomics), community scale microbiota composition of ileal digesta (Microbiota), 24 different cytokine levels in blood serum (Cytokine), and protein-associated metabolic profiles of serum (Metabolomics Serum) and urine (Metabolomics Urine). These data were pre-processed and analyzed separately by fitting a linear model on the data-points and looking for differentially expressed readouts in each treatment vs. the control. Each dataset had its own *p*-value (corrected for multiple testing with the Benjamini–Hochberg method) threshold, ranging from 0.001 to 0.1 for difference between the tested and reference diets. The highest number of statistically significant entities was found in Transcriptomics. Furthermore, all the measured variables in Metabolomics Urine were found to be significantly different in at least one comparison.

### Pairwise data association and network generation

**Figure 2:**
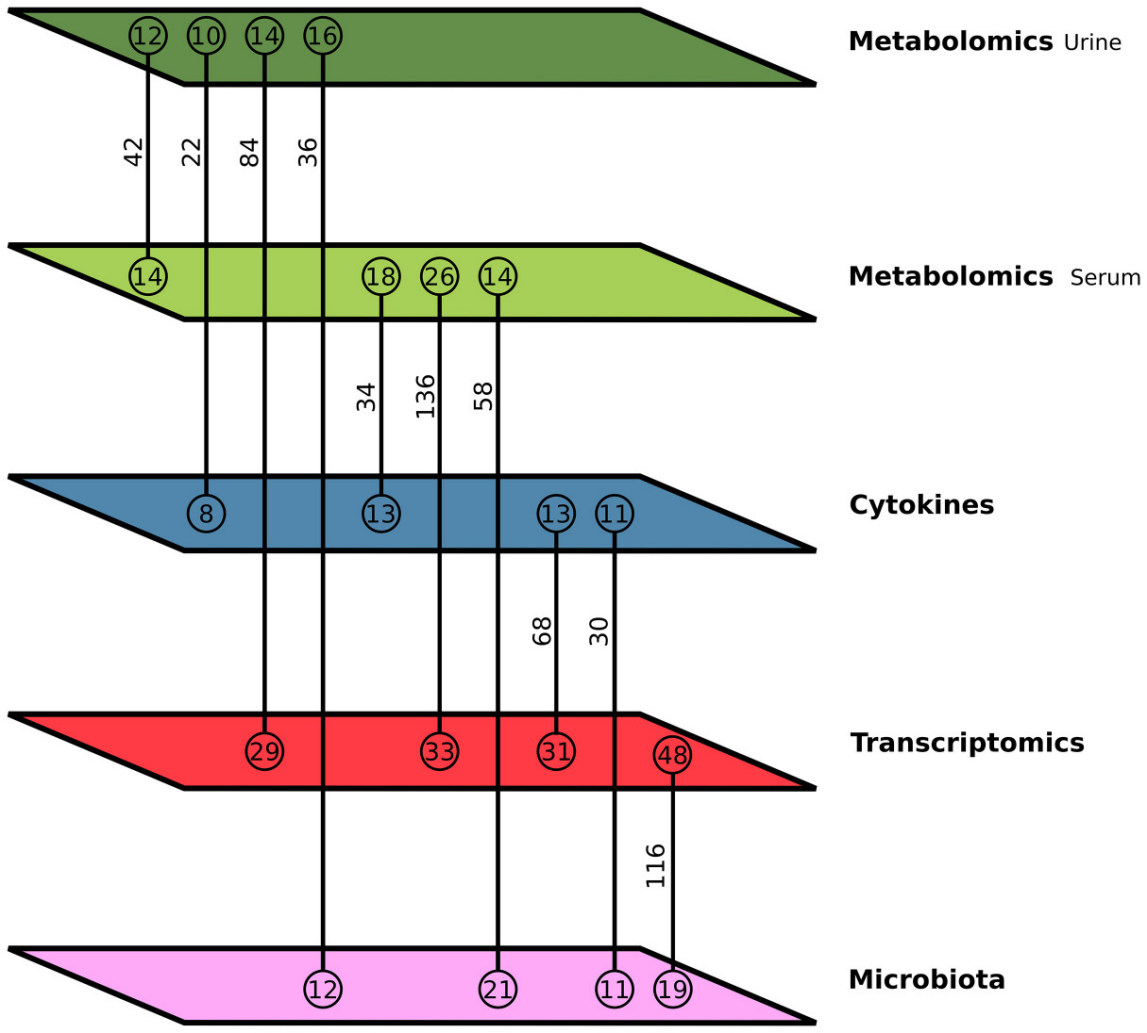
Multilevel integration. This schematic image shows the number of connections between each internal phenotypic level with the other levels in a merged network. The colors of the parallelograms denote the internal phenotypic level to which the data-types belong. Green is Metabolomics (light green—Metabolite from Serum and dark green—Metabolite from Urine), blue is Cytokines, red is Transcriptomics, and pink is Microbiota. Each line connects two levels and the vertical number above the line indicates the number of edges in the correlation network between those two phenotypic levels. The number of connected nodes in each level is given in circles above and below the connecting lines.

We performed the integration by linking two data-types at a time and in such a way that after the pairwise analysis all the observed association data could be combined to build a multi-level interaction network. Therefore, each data-type was integrated with the other four types of data, resulting in 10 association networks. The topological characteristics of all these 10 networks are given in Table 2 and Figure 2, and the network graphs are available in Supplementary Figure 2 as an image. Data Sheet 1 has the networks in a format that can be uploaded into Cytoscape in order to further explore the connectivities of these networks by simply clicking on these nodes. Table 2 shows the positive and negative thresholds that were used separately for the association network. Connections between pairs of data points with correlation values between the threshold values, i.e., Low Threshold (negative threshold) and High Threshold (positive threshold) as indicated in Table 2, were discarded and the corresponding edges removed from the final network. There were two disconnected sub-graphs in five of the networks while the other five have only a single, fully connected graph. Supplementary Figure 3 shows the pattern of changes induced by the diet in three components of the network Microbiota & Transcriptomics.

**Table 2:** The 10 individual correlation networks.

**Network names (data A & data B)**	**Low threshold**	**High threshold**	**No. of nodes (A)**	**No. of nodes (B)**	**Connected components**
Metabolomics Serum & Metabolomics Urine	−0.51	0.6	14	12	2
Metabolomics Serum & Microbiota	−0.38	0.3	21	14	1
Metabolomics Serum & Transcriptomics	−0.31	0.35	26	33	2
Metabolomics Serum & Cytokine	−0.33	0.5	18	13	2
Metabolomics Urine & Microbiota	−0.28	0.42	16	12	1
Metabolomics Urine & Transcriptomics	−0.55	0.54	14	29	1
Metabolomics Urine & Cytokine	−0.32	0.55	10	8	2
Microbiota & Transcriptomics	−0.28	0.27	19	48	1
Microbiota & Cytokine	−0.38	0.35	11	11	1
Transcriptomics & Cytokine	−0.27	0.34	31	13	2

*Each row represents one of the 10 correlation networks. Low Threshold and High Threshold represent the thresholds used for the correlation values. The 3rd and 4th columns have the number of nodes in the network that belong to the first and second data, respectively. The last column displays the number of connected graphs in the network*.

The largest network, in terms of nodes, is the Microbiota & Transcriptomics network. This seems logical as it represents the most comprehensive datasets and spacial interactions between the two data-types are known to occur. Overall, networks involving Transcriptomics data had higher number of nodes than other networks. The smallest network with 18 nodes and 22 edges was the Metabolomics Urine & Cytokine network.

### Technical validation of pairwise integration networks by random permutation

**Figure 3:**
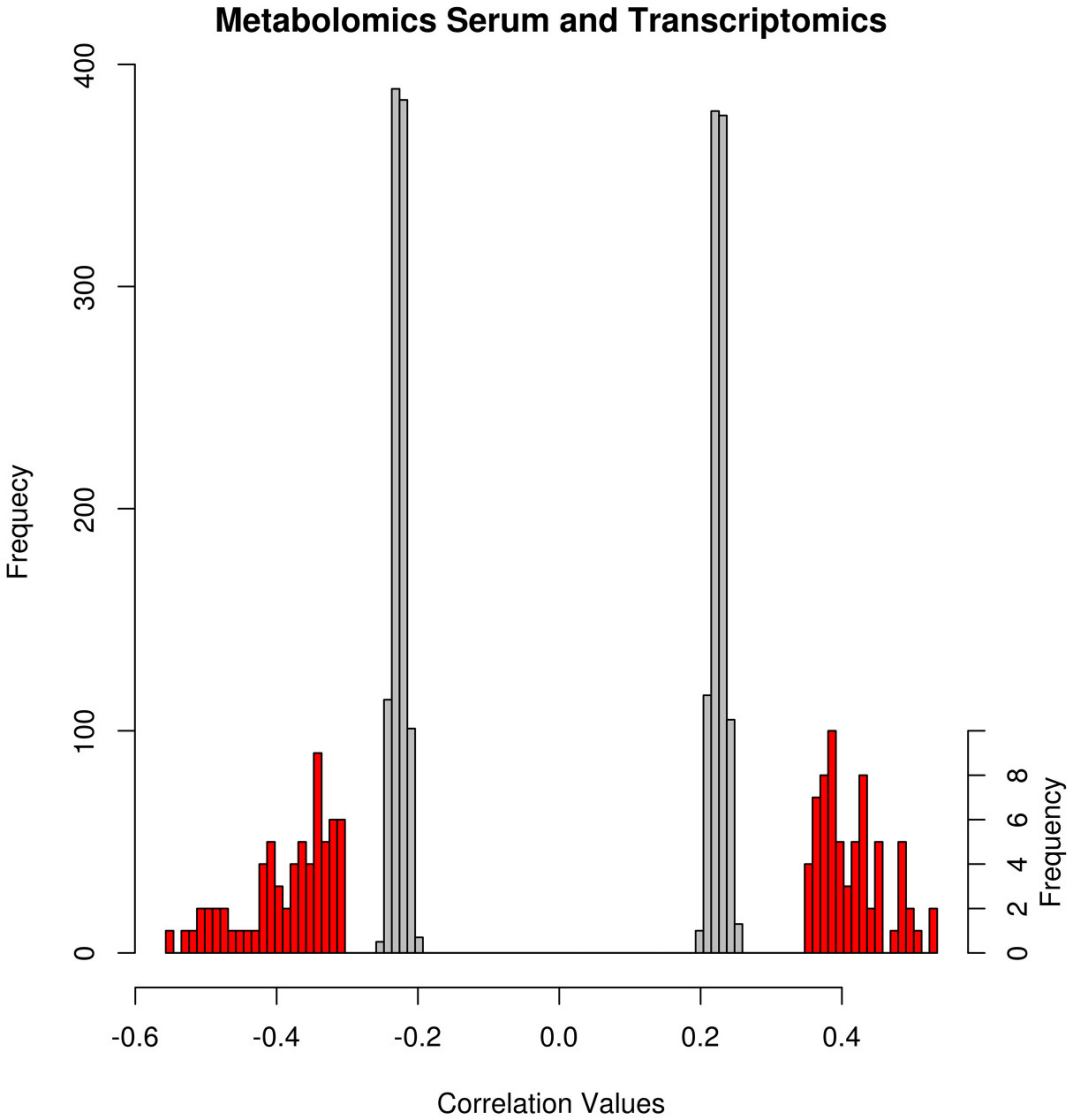
Distribution of network correlations and random network cut-offs of the Metabolomics Urine and Transcriptomics networks. The x-axis depicts the range of correlation values and the y- axis shows its frequency. The gray bars denote the distribution of the thresholds of the 1,000 random correlation networks with frequency on the left y-axis. The red bars are distributions of the correlation values of the inferred network with frequency on the right y-axis.

We performed the same method of integration on the five different data-types after randomly permuting the measured data, this process was iterated a 1,000 times. In this way, the networks obtained from random permutations are considered a null model with no biological information, and used to assess the significance of the results obtained with the non-permuted data. Figure 3 shows the spread of correlation values for the integration of Metabolomics Serum and Transcriptomics. The thresholds for network reconstruction were selected so that only the 5% highest and lowest correlations were kept. The separation between the values obtained for the integrated data and the randomly permutated datasets indicates the high significance of the edges in the integration networks. In this way, selection of the 5% highest and lowest correlations and significant limits the number of spurious correlations that could be due to chance alone while retaining maximum information in the networks.

Similar results were obtained for most of the integration networks (Supplementary Figure 3). In three of the networks, there is an overlap between the correlation values from the inferred network and the values arising from the randomly generated networks. The overlaps are in the networks Metabolomics Urine & Microbiota, Metabolomics Urine & Cytokine, and Transcriptomics & Cytokine network. The highest overlap appears in the first two and mostly affects edges with negative correlations.

### Merged network

All the 10 integration networks (Data Sheet 1) were merged and only nodes linked with nodes of at least two other data-types were kept (see Table 3). The gene expression data has the highest number of nodes in the merged network. However, nodes with the highest degree (number of connecting edges) arise from the microbiota data, with S24-7 having 57 neighbors and Bifidobacterium having 47 neighbors. The merged network encompasses 45 nodes that are connected to all the other types of data. For that reason we denote them “Connectivity hubs” and they are included in Table 3 and Supplementary Table 1.

**Table 3:** Characteristics of the merged network.

**Network statistics**	
Total number of nodes	112 (45)
Total number of edges	577
Number of Metabolomics Urine nodes	15 (8)
Number of Metabolomics Serum nodes	24 (11)
Number of Cytokine nodes	13 (7)
Number of Transcriptomics nodes	43 (12)
Number of Microbiota nodes	17 (7)
Degree range	2–57
Average number of neighbors	10.35
Clustering coefficient	0.20
Characteristic path length	2.31
Network density	0.09
Connected components	1

*Characteristics of the merged correlation network. The number of nodes from each data-type are given in rows three to seven. Between brackets the number of connectivity hubs is indicated*.

### Functional validation of merged network by text mining

A PubMed literature search for co-occurrence of linked nodes gave results for 6% of the links corresponding to 37 edges. We further investigated reported causality effects between the nodes in question. Most of the retrieved results are related to metabolites and cytokines measurements whereas a few results confirming causal relationships were found involving gene nodes. We were able to find literature confirmation pertaining to associations for six out of the 10 pair-wise connections between phenotypes, as summarized in Table 4. Supplementary Table 2 contains all the PubMed identifiers from the literature mining and Supplementary Table 3 has phrases from a maximum of three PubMed abstracts from the results. Among the nodes with literature results, four are from Microbiota, two from Transcriptomics, 15 from Metabolomics Serum, three from Metabolomics Urine, and six from Cytokines. The node with the highest number of hits in literature is Tnfa which co-occurs 8,563 times with nine metabolites from the Metabolomics Serum data and one bacterial group (Bifidobacterium).

**Table 4:** Overview of text mining results.

**Data connections**	**PubMed Ids**	**Distinct edges**
Cytokines & Metabolomics Serum	9,554	16
Metabolomics Serum & Metabolomics Urine	906	6
Microbiota & Metabolomics Serum	254	7
Microbiota & Cytokines	250	5
Transcriptomics & Microbiota	83	3
Metabolomics Serum & Transcriptomics	59	2

*The first column shows the types of data that are connected by the edges that were found in the PubMed literature search*.

**Figure 4:**
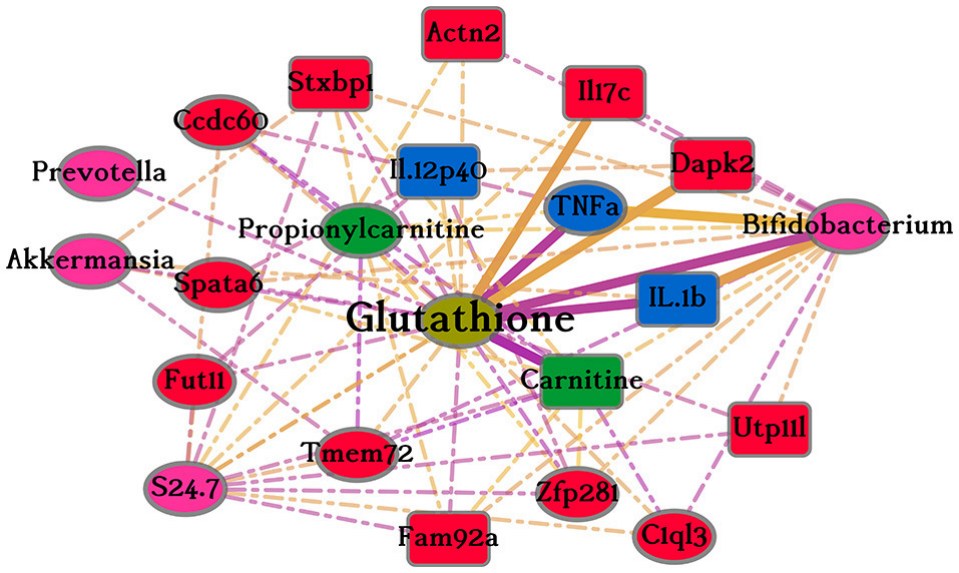
Glutathione sub-network. This figure shows the 21 connections of the node Glutathione in the merged network. The different colors of nodes indicate the data-type of internal phenotypic level of that node, pink is Microbiota, red is Transcriptomics, blue is Cytokines, and green is Metabolites (light green—Metabolites from Serum and dark green—Metabolites from Urine). Oval nodes are connectivity hubs. Dotted lines show un-validated edges and continuous, thicker edges show connections also present in the results retrieved from scientific literature. Edge color, yellow and purple, indicates positive and negative correlations, respectively.

Of the 30 data-points from all the types of data that have literature results, 15 are connectivity hubs. One such connectivity hub is Glutathione (GSH) which has 21 direct neighbors from four data-types as shown in Figure 4. This hub is especially interesting because six of the connected nodes (Carnitine, Tnfa, Il-1b, Il17c, Bifidobacterium, and Dapk2) have textual co-occurrences found by the text mining algorithm. The terms GSH and Tnfa were found 2,231 times in the abstracts of Pubmed indexed articles. Full text inspection shows that some of the connections are causal relationships as one of the connected nodes activates or inhibits the other.

## Discussion

In this study we developed and used a set of computational methods to identify components in internal phenotypic layers that are connected to components in other internal phenotypic layers of an organism. We successfully integrated multi-scale quantitative (-omics) data, derived from a single experiment with inbred mice and which were exposed to five different diets. Here the mice had been exposed to the dietary intervention for 4 weeks. Four weeks is a significant amount of time in the life of mice and previous studies comparing the development of mice and humans (specifically the immune system in Holladay and Smialowicz, 2000) indicate that the development of different systems is much faster in mice than in humans. Hence it is reasonable to assume that the mice have adapted to the new diet in 4 weeks. Since the data originated from an animal experiment that was not designed for the detection of genetically and/or dietarily induced differences in external phenotypes, we only focused on the connectivity between 5 intermediate phenotypic levels. Some studies have reported pairwise data integration of two (Lu et al., 2014; Rajasundaram et al., 2014; Benis et al., 2015) or three data sets (Adourian et al., 2008). But this is, to the best of our knowledge, the first time that an integration of such heterogeneous data-types from different tissues, arising from a single experiment, has been reported. The approach as described here could, in principle, be applied on any number and type of datasets, as long as they are from the same experiment, from samples at the same time-point and have comparable dimensions of differentially regulated data.

### Internal phenotypic data and pairwise data integration

Each used data-type represents a different internal phenotype and a different layer of the system that (co-) drives the manifestation of external phenotypes. We subjected each data-type to a separate analysis in order to correlate only those changes induced by the dietary intervention. Nodes with significantly different values could easily be identified in each of the sampled tissues and fluids (ileum, blood, and urine) thereby representing the local and systemic effects of the interventions and the need of a multi-scale approach.

In order to investigate connections between the five data-types we used sPLS, an integration method that can be applied to several types of data, two at a time. This method can also handle the dimensionality problem of biological datasets where the number of variables is usually higher than the number of samples. sPLS has been previously used for integration of microbiota with gene expression data (Benis et al., 2015; Steegenga et al., 2016), and measurements on cell wall polysaccharides of fibers with phenotypic characterizations of fibers in cotton balls (Rajasundaram et al., 2014).

We performed pairwise integration of the datasets, resulting in 10 networks with varying spreads of correlation values. Deciding on a threshold to distinguish genuine from spurious correlations is a major bottleneck for the definition of association networks. While a 0.8 threshold (absolute value) has been suggested for gene expression data (Schäfer and Strimmer, 2005), other authors suggested smaller values (0.6) in metabolomics data sets (Camacho et al., 2005). The correlation values greatly depend on the biological dataset under study and its dimensionality. There are several methods to choose a threshold based on the data: use assigned *p*-values as threshold; use network characteristics of the correlations; or use a percentage of the correlation distribution. When evaluated by Borate et al. (2009) they concluded that threshold selection methods based on network properties such as the clustering coefficient are best for gene co-expression networks. This would not work here because the generated networks always induce connections between data points of different type and as a result they have a zero clustering coefficient for every node. While integrating two types of metabolomics datasets with gene expression of the tissues in which they were measured Adourian et al. (2008) assigned *p*-values to the correlation values and then set a threshold. Selecting a threshold is further complicated by the possible appearance of spurious correlations due to a common response variable influencing the connecting nodes (A is correlated to B, A is correlated to C, therefore, B and C appear correlated). Regarding gene expression data, multiple methods (reviewed for example in Marbach et al., 2012) have been developed to minimize the number of falsely predicted associations. In this study, we used the top 5% of the correlation values because this dynamic threshold (separate for the positive and negative values) eliminates bias toward the size of the datasets. To further evaluate the impact of the correlation scores we have inspected the correlations between some linked nodes. Supplementary Figure 3 shows an extreme case in which transcript abundance of two genes negatively correlated with the abundance of a bacterial group. This might induce a spurious association between the genes. Spurious associations due to a common response variable influencing the connecting nodes are more likely to appear when both nodes are of the same type. Therefore, to further minimize the number of spurious associations we have focused on associations between different internal phenotypes.

We further validated the observed correlations by comparing them with a null model obtained by randomly permuting the data along the samples (Eguíluz et al., 2005; Saccenti et al., 2015). In the randomly permuted samples we expect all inferred associations to be spurious, as the permutation process destroys any possible correlation between the variables. In that case, even the correlations corresponding to the highest and lowest 5% of the population would be spurious. The values of the correlations deemed significant in the experimental data sets are found to be higher than these false positives. In two of the networks, Metabolomics Urine & Microbiota and Metabolomics Urine & Cytokine (the smallest network), the significance of the negative correlation values could not be established as we observed a considerable overlap between the negative correlation values of this network and the negative thresholds of the random networks. This calls for caution when biologically interpreting these networks. For five of the networks we observed a very clear separation of the random thresholds and the start of the correlation values in the network (Supplementary Figure 4). The other networks showed slight overlaps between the random threshold distribution and the network correlation distribution. This extra validation step reassured us that the observed correlations are rooted in biological phenomena. To our knowledge this technical validation step is not common in current studies of this type.

The edges of the inferred networks, indicate significant computationally-determined correlations between values of connected nodes. Our approach does not require a mechanistic model on how the associations are established and in each network these associations may be caused through entirely different mechanisms. In some cases the associations would be due to causal relationships between the connected nodes, such as increased expression levels of a cytokine gene linked to increased cytokine levels. However, in many cases, the associations could be indirect, mediated by elements that have not been measured in the experimental set up. In a formal mathematical model, they are considered hidden variables. Such would be the case of, for example, the changes in the metabolite levels of urine. These changes might have been caused by the colonic microbiota, in turn affected by the ileal microbiota. Since we only used the ileal microbiota data, we observe correlations between the ileal microbial populations and the urine metabolite levels which could be in reality, indirect relationships mediated by the colonic microbiota.

### Network of connected internal phenotypes

The pair-wise integration method allowed us to merge the 10 individual networks into a single network. Correlations within a dataset were deliberately excluded from this study because we only wanted to focus on connections between different internal phenotypes, where little work has been done. Thus, in the 10 networks, all detected connections are between two different data types and every node has a zero clustering coefficient. However, in the merged network, a non-zero clustering coefficient emerges as a result of nodes connecting to multiple data types (Table 3). This emphasizes the biological relevance of this method because the 10 networks were built without any information on cross-linking. Thus, we identified individual nodes that directly or indirectly participate in processes of the other four individual networks. Because they seem to connect different internal phenotypes, we denoted them “Connectivity Hubs.” Starting the procedure as developed and applied here with networks with non-zero clustering coefficients (correlating within a dataset) would, however, not alter the connections between internal phenotypes.

### Functional validations of phenotype connections

Results of the text-mining were used to validate some of the identified links. This revealed insights into the mechanistic relationships between the variables predicted to be linked to each other. Thirty-seven of the 577 (6%) computational inferred links have already been described in literature as detected by our text-mining approach, which was not exhaustive because it focused only on text in journal abstracts. This indicates that our method identifies currently known biological interactions. The rest of the predicted links have not been discovered and investigated yet, have not been mentioned in abstracts, or do not exist in the biological system. Furthermore, by inspecting some of the retrieved abstracts and corresponding articles, we were even able to find causal relationships between some of the computational identified nodes where one of the nodes was used as an experimental perturbation and the other node was measured as a response parameter. Some examples are shown in Supplementary Table 3. Several indirect associations were also validated through reports on experiments where nodes, found to be connected in this study, were measured in response to another perturbation. During text-mining, in order to retrieve as many results as possible, search terms were matched against the MeSH thesaurus, irrespective of the organism, and all the synonyms were included in the search. The downside to this approach is the inclusion of several false textual associations. The most striking case is that of the identified association between Glutathione and Il17c. In the literature results, the reported association is between Glutathione and Il17a and not Il17c. Through the thesaurus, Il17c was mapped to Il17 and subsequently to Il17a thereby giving rise to that falsely identified association in literature.

In order to increase the precision and recall of text mining searches, and overcome problems associated to the use of a thesaurus, one needs to move from mining text, to mining the knowledge embedded in the text and the use of data hidden in public databases. Such an approach requires the use of knowledge management tools and representations that can be automatically accessed (Antezana et al., 2009). Semantic web technologies represent a new class of tools that include natural language processing, ontologies, machine learning algorithms and much more to facilitate integration knowledge from heterogeneous sources. The expansion of the use of semantic technologies in the life sciences domain will allow associating concepts such that inferences on causality, regulation, organism, or tissue can be made using high-throughput methods and automated reasoning.

Among the interactions retrieved from the automated literature search, a high prevalence of associations involving cytokines and/or metabolites was observed. In fact, such type of interactions represent 97% of the retrieved results. This probably highlights the extraordinary amount of work that has been done in these types of data in the past. On the opposite extreme, only 8% of the retrieved interactions involved associations between the expression of genes, reflecting the fact that most of the available gene expression data originates from genome-wide techniques. In such type of experiments, papers, especially abstracts, usually report on systems behaviors and pathways and less frequently on the individual behavior or role of individual genes and connected response nodes.

### Validated connectivity hubs

Even though we only performed integrations of two datasets at a time, we find data-points (metabolites, cytokines, genes, or microbial groups) that correlate with different types of data. We identified 45 connectivity hubs in the merged network that seem to have associations with all four types of data. More than 30% of them are involved in links that were retrieved in literature. To further support the biological relevance of identified multi-level connectivities we discuss the implications of two of the 15 biologically validated connectivity hubs as examples. The two connectivity hubs were chosen because of the large amount of literature results for these hubs. The first hub, Tnfa has the highest number of literature results among all the nodes in the network and the other hub, Glutathione, has literature validations to the most number of data-types.

Tnfa is a connectivity hub in the merged network, with links to several neighbors belonging to the four other types of data. The position of this cytokine in our merged network shows that it plays a role in processes of the other internal phenotypes. The literature validated links are between Tnfa and two other types of data (Metabolomics Serum, Microbiota). Many of the validated links represent causal relationships. With regards to immune responses and as a drug target, Tnfa has been studied in great detail (Cicha and Urschel, 2015). The un-validated edges show that Tnfa could be a regulator of other internal phenotypes as well, than currently known.

The metabolite Glutathione (GSH) was measured in the serum and in the merged network is a connectivity hub proving that it is vital part of the system that connects several internal phenotypes. Among the 15 connectivity hubs with functionally validated links, GSH is the only one that has validated links to all other data-types based on our literature mining. These results support our claim of GSH being a connectivity hub, a biological component influencing several internal phenotypes. Several PubMed results for GSH are from *in-vivo* studies where GSH was administered to alleviate symptoms of a disease. Our literature results show that GSH has been studied in relation to all different types of data. Of the six validated links in our merged network, five represent proven causal relationships (see Figure 4 and discussion of the functional validation). These neighboring nodes in the merged network are mostly related to immune and homeostatic mechanisms. GSH is a tripeptide, ubiquitously distributed in living cells and plays an important role in the intracellular defense mechanism against oxidative stress (Diaz-Vivancos et al., 2015; Couto et al., 2016). It is known that GSH metabolism is very important for the antioxidant and detoxifying action of the intestine. It is also essential for the maintenance of the luminal thiol-disulfide ratio involved in regulation mechanisms of the protein activity of epithelial cells (Iantomasi et al., 1997) which could be important since the intervention is changes in protein. Our results also demonstrate the manifold and central role of GSH when it comes to proteins, peptides and amino acids in nutrition. These observations indicate that the presented merged network represents, at least in part, associations of biological phenomena.

### Potential relevance of selected connectivity hubs

There are 30 connectivity hubs in the merged network that do not co-occur with their connected nodes in our literature search. However, the prominence of these nodes in our merged network indicates that they could represent potential relevant interactions with components of the other internal phenotypes. In order to demonstrate how the results of this study may be used to hypothesize on functional relationships between different molecular components, we here describe the potential biological relevance of two highly linked connectivity hubs, Tmem72 and S24-7. Both hubs are not yet described in literature abstracts in conjunction with other data-types.

The high number of connectivity hubs in the Transcriptomics layer suggest that the expression of several intestinal genes is involved in many more interactions than currently known. None of the observed Transcriptomics connectivity hubs popped-up in our literature mining results. The most highly connected Transcriptomics node, Tmem72 (Transmembrane Protein 72), has only been studied in the kidney so far (Habuka et al., 2014) and not much information is available on it. But in the merged network this node has 27 links to other data-types (can be visualized in Data Sheet 1), mostly to metabolites from both the metabolomics datasets. Based on this, we hypothesize that Tmem72 is not specific to the kidney and that it has some sort of communication function in intestinal mucosa as well. The fact that Tmem72 is a transmembrane protein is supportive for this. Given its observed links with different microbiota, metabolites, and cytokines, it might be involved in diverse interactions with other internal phenotypes. Based on such an hypothesis, targeted experimental designs may be developed in order to investigate the hypothesized “communication” function of Tmem72 in intestinal mucosal tissue.

The most highly linked node of the merged network is the bacterial family classification, S24-7, suggesting an important role for this species in gut functionality. In some of the inferred individual association networks we already found it to be linked to a high number of nodes. Unfortunately, this node is not represented in literature abstracts together with the here observed neighbors. However, there is compelling literature that shows this microbial classification to be a significant part of the gut microbial community structure (Harris et al., 2014; Jakobsson et al., 2015). This family classification does not have a good functional definition, yet several studies show that it could be an important player in the functionality of the gut (Evans et al., 2014; Harris et al., 2014; Rooks et al., 2014). The latter claims are in line with the high number of neighbors that S24-7 has in our merged network. The current technical inability to cultivate S24-7 is most certainly due to the absence of knowledge on S24-7 interactions. However, a recent *in-silico* study (Ormerod et al., 2016) shows that S24-7 species have the ability to survive on different types of carbohydrate sources, similar to the genus Bifidobacteria. In the merged network, the connectivity hubs S24-7 and Bifidobacteria, share the highest number of neighbors (directly linked nodes). Among them are 16 genes, and neither S24-7 nor Bifidobacteria have literature results with any of these genes. An enrichment analysis on these shared network gene neighbors shows that they are involved in functions related to linoleic and linolenic acid metabolism (data not shown). It is known that these fatty acids are produced by Bifidobacteria (Teran et al., 2015) and that they are involved in the maintenance of the epidermal barrier function (Muñoz-Garcia et al., 2014). The observation that in our network these genes are shared between S24-7 and Bifidobacteria underscores the here hypothesized importance of S24-7 and indicates that these two bacterial groups are indeed closely related in function as hypothesized before (Ormerod et al., 2016).

From the results described in this paper, we conclude that we successfully developed methodologies to identify components in internal phenotypic layers that are connected to components in other internal phenotypic layers. By integrating multi-scale quantitative (-omics) data using a regression approach, we were able to provide provisional insight into potential ways internal phenotypic layers are connected to each other, including those between local and systemic layers. By a technical and functional validations, we underscored the relevance of our findings. Based on data generated by this type of integrated approaches, hypothesis driven and targeted research may be developed to identify causal relationships between various biological scales in order to diminish our knowledge gap between genotype and external phenotype. In addition, by expanding comparable approaches by incorporating data on genetic diversity and/or variation in external phenotypes, this knowledge gap may be even further closed down. The analysis pipeline that we developed is very general. Here we demonstrated this pipeline with datasets that address only one of the multiple environmental factors that might affect the internal phenotypes, namely the diet. However, the approach is very general and can be adapted to any type or number of data sets describing the impact of other perturbations.

## Availability of data and materials

Transcriptomics data has been uploaded into GEO with the accession number GSE84442. The microbiota data, the two metabolomics datasets and the cytokine data are available on request. The R scripts using functions from existing R packages are also available on request.

## Author contributions

NB performed the data analysis and prepared the manuscript. SK performed the animal experiment and contributed significantly to the biological interpretation of the results. VM contributed to the direction of the analysis and the manuscript. MS was involved in the animal experiment and helped with the direction and critical revision of the manuscript. DS was involved in the data analysis and biological interpretation. MS-D helped with the direction of the data analysis and the manuscript. All authors have read and approved of the final manuscript.

### Conflict of interest statement

The author VM was employed by company LifeGlimmer GmbH. This author contributed to the data analysis and the writing of the manuscript. All other authors declare that the research was conducted in the absence of any commercial or financial relationships that could be construed as a potential conflict of interest.
