# Supplementary material for: Multi-Level Integration of Environmentally Perturbed Internal Phenotypes Reveals Key Points of Connectivity between Them
Source: Front Physiol. 2017 Jun 12;8:388. doi: 10.3389/fphys.2017.00388 (PMC5467433; doi:10.3389/fphys.2017.00388)
Supplement: Supplementary file 3 [file Table3.DOCX]

| **Associated data points** | **Pubmed Id** | **Connecting phrase in abstract** |
| --- | --- | --- |
| TNFa AND Tryptophan | 26057461 | The negative effects of **Trp (dietary Tryptophan)** deficiency on those tight junction protein gene expression might be partly related to the increases in the mRNA levels of pro-inflammatory cytokines and related signalling factors (**tumor necrosis factor α**, interleukin 8, interleukin 1β and transcription factor-κB) |
|  | 19428234 | **tryptophan** reduced the expression of the pro-inflammatory cytokines tumor necrosis factor-alpha, |
|  | 18812628 | **TRD (tryptophan rich diet)** exerted opposite effects and significantly accelerated ulcer healing. This last effect was accompanied by significant decrease of **TNF-alpha** mRNA expression and expression of NFkB-p65 in gastric mucosa |
| IL.6 AND Valine | 25558818 | γ-EC and γ-EV (gamma glutamyl **valine**) reduced the expression of TNF-α, **IL-6**, INF-γ, IL-1β, and IL-17, |
|  | 24870967 | Real-time PCR analysis revealed that the expression of monocyte chemoattractant protein-1 and that of the pro-inflammatory cytokine **IL-6** in adipose tissue tend to be lower in the HFD+VPP (high fat diet with **valine**-proline-proline) group than in the HFD group |
|  | 23324083 | LRP most efficiently neutralized the LPS-induced pro-inflammatory mediators like NO, TNF-α, and **IL-6** in macrophages followed by FRP, VRP (**valine** residue peptide), and ARP. |
| Il17c AND Staphylococcus | - | - |
| Glutathione AND TNFa | 26742325 | decreased **glutathione** levels, superoxide dismutase and catalase activities) in liver tissues and inflammatory surge (serum **TNF-α**) significantly |
|  | 26634045 | Treatment with EAF (ethyl acetate fraction) resulted in significant suppression of oxidative stress in RAW264.7 macrophages as demonstrated by increased endogenous superoxide dismutase (SOD) activity and intracellular **glutathione** levels […] To confirm its anti-inflammatory effects, analysis of expression of inducible NO synthase, cyclooxygenase-2, **tumor necrosis factor-α**, […] was performed using semi-quantitative RT-PCR. EAF treatment resulted in significantly reduced dose-dependent expression of all of these factors, |
|  | 26161237 | Obstructive jaundice led to a significant increase in the serum total bilirubin, AST, and ALT levels. The proinflammatory cytokines levels (**TNF-α** and IL-1β), concentration of NO, and oxidative stress markers (MDA and 3-NT) were increased as well. All of those were reduced by the treatment of GSH (**glutathione**). |
| Glutathione AND IL.1b | 27036629 | Additionally, KGLY (Kegan Liyan oral liquid) markedly attenuated LPS-induced acute pulmonary inflammation via decreasing the expressions of cytokines and chemokines (**IL-1β**, IL-6, TNF-α, and MIP-2), enhanced the activities of anti-oxidative indicators (SOD and **GSH** **(glutathione)**) |
|  | 27023766 | Mice anesthetized with isoflurane and sevoflurane showed thinner alveolar septa, lower VILI scores, lower polymorph neutrophil counts, and lower **interleukin-1β** concentrations than ketamine mice. Mice anesthetized with isoflurane and sevoflurane showed less ROS production and higher **glutathione** contents compared with ketamine mice. |
|  | 26728795 | Se deficiency reduced SIgA amount in the duodenal mucosa but increased the level of interleukin-1β (**IL-1β**), IL-17A, tumor necrosis factor-α (TNF-α), and interferon gamma (IFN-γ). Additionally, Se deficiency increased oxidized **glutathione** activity, whereas decreased glutathione peroxidase and glutathione activities (P < 0.05), |
| Glutathione AND Il17c | - | - |
| Glutathione AND Dapk2 | - | - |
| Glutathione AND Carnitine | 26888052 | L-**carnitine** in low and high doses (50 and 500 mg/kg) was administered for five consecutive days to male Wistar rats. Hepatocytes were isolated and freshly exposed to appropriate concentration of T-2 toxin for 2 h followed by oxidative stress and cell death evaluations. […] **Glutathione** depletion, ROS overproduction and mitochondrial membrane potential collapse were determined under T-2 toxin exposure. |
|  | 26804544 | Based on available data, it seems that BC metabolic signature is mainly characterized by alterations in metabolites related to energy metabolic pathways, particularly glycolysis, amino acid and fatty acid metabolism, known to be crucial for cell proliferation, as well as **glutathione** metabolism, known to be determinant in maintaining cellular redox balance. In addition, purine and pyrimidine metabolism as well as **carnitine** species were found to be altered in BC. |
|  | 26701137 | Malondialdehyde values of all of the L-**carnitine** groups were significantly lower than those of the H2O2 group, while total **glutathione** levels of all of the L-carnitine groups were significantly higher than of the H2O2 group. |
| Kynurenine AND TNFa | 23606516 | The results show that the in vivo LPS administration induced increased plasma concentrations of **TNF-α** and IL-10, a depletion of Trp and an increase of **Kyn** **(kynurenine)**, indicating an elevated enzymatic activity of IDO. |
|  | 23201589 | Poly I:C induced a neuroinflammatory response characterised by increased expression of IL-1β, IL-6, **TNF-α** and CD11b in frontal cortex and hippocampus. In addition, poly I:C increased central IDO expression and increased concentrations of tryptophan, and its metabolite **kynurenine**. |
|  | 21819405 | The results show increased levels of tryptophan with decreased levels of **kynurenine**, anthranilic acid and 3-hydroxyanthranilic acid associated with bypass, and a later increase in kynurenic acid. Levels of neopterin and lipid peroxidation products rose after surgery in non-bypass patients whereas **tumour necrosis factor-α** and S100B levels increased after bypass. |
| Citrulline AND TNFa | 25320354 | Therefore, we hypothesized that L-arginine or L-**citrulline** supplementation would ameliorate diabetic nephropathy. Nonsupplemented diabetic mice showed significant increases in albuminuria, blood urea nitrogen, glomerular histopathological changes, kidney macrophage recruitment, kidney **TNF-α** |
|  | 24360391 | No significant correlations were found between **citrulline** and albumin, transthyretin, **TNF-α**, IL-10, or TNF-α/IL-10 ratio. |
|  | 23514809 | Elevated expression of proinflammatory cytokines [interleukin-1β (IL-1β), IL-2, interferon γ, and **tumor necrosis factor-α**] and the transcription factors [Janus kinase 3 (Jak3) and signal transducer and activator of transcription 3 (Stat3)] was found in the 1,2-dimethylhydrazine dihydrochloride (DMH) group; [...] Expression of inducible nitric oxide synthase and nitric oxide/**citrulline** levels was also analyzed and was found to be elevated with DMH treatment. |
| Arginine AND TNFa | 25686746 | the mRNA levels of HMGB1 were remarkably increased at the 12 h, peaked at 24 h, and remained at a high level up to 48 h after L-2 **arginine** injection. The levels of **TNFα** and IL-6 were decreased at 48 h |
|  | 25501750 | L-ARG (l-**arginine**) protected against myocardial cellular death by reduction in NFκ-B mRNA as well as **TNF-α** level |
|  | 25443726 | plasma concentration of l-citrulline (as a marker of NO production from l-**arginine**) … Omentin-1 significantly increased l-citrulline levels in plasma (p<0.05), and the gene expression of adiponectin in PAT (p<0.05). On the other hand, we found decreased gene expression of IL-6 (p<0.005), while **TNF-α** mRNA in PAT was not affected. |
| Glutamine AND IL.2 | 26734536 | as well as **IL-2** plasma levels normalised faster in patients who received **glutamine**-supplemented diets than in patients who received isocaloric, isonitrogenous diets |
|  | 23922725 | 3 amino acid treatments fed 1.0% arginine (Arg), 1.0% **glutamine** (Gln) and 0.5% Arg+0.5% Gln, respectively, … The **IL-2** and TNFα values in the amino acid groups were similar to those in non-toxin control, and significantly lower than those in toxin control (P<0.01). |
|  | 23583300 | tBHQ and 5μM B[a]P (benzo[a]pyrene) caused similar alterations of **IL-2** secretion and **glutamine**/glutamate metabolism. |
| Aspartic.acid AND IL.6 | 25153417 | glutamic acid and **aspartic acid** decreased during surgery while asparagine increased. Jugular vein **interleukin (IL)-6** showed a transient non-significant increase during clamping and decreased systemically. |
|  | 9189931 | we measured the levels of glutamine, **aspartic acid**, glutamic acid and GABA in the hippocampus and hypothalamus of mice treated with **IL-6**. At both doses which affected the cognitive functions, this cytokine had no effect on brain levels of measured amino acids. |
| Aspartic.acid AND IL.2 | 21062264 | One of the most effective amino acid mixtures was found to be leucine, **aspartic acid** and glycine. This amino acid mixture was utilized for the production of **IL-2** in batch and fed-batch fermentations. |
|  | 19341362 | Glutamine, a mixture of leucine, **aspartic acid** and glycine, and a mixture of leucine, glutamine and aspartic acid, were the most effective for the expression of **IL-2**. |
| Threonine AND TNFa | 25923079 | In LPS treated spleens, **TNF-α** expression was also up-regulated by NanoAg, amino acids (cysteine, **threonine**) and their combinations, |
|  | 24249671 | Tipα (**tumor necrosis factor-α** -inducing protein) enhanced the phosphorylation of 11 cancer-related proteins in serine, **threonine** and tyrosine, indicating activation of MEK-ERK signal cascade. |
| Alanine AND TNFa | 7630167 | **TNF alpha** administration resulted in a 50% increase in gluconeogenesis from **alanine** (P < 0.05) |
|  | 8651752 | Both IL-6 and **TNF-alpha** exerted a small stimulatory effect on **alanine** and glutamine transport. |
|  | 8778147 | Six hours after administration of the **TNF**, the total skeletal muscle amino acid concentration was significantly reduced by 20%. The greatest reductions were seen in lysine, arginine, and isoleucine (39-53%) followed by serine, tyrosine, ornithine, threonine and **alanine** (18-32%). |
| Alanine AND Acetylcarnitine | 26317529 | **Acetylcarnitine** (C2), showing a late response pattern and having the highest values in MFC and statistical significance, was classified as late marker and ranked as strong predictor (MFC = 1.97, P < 0.001). In the class of amino acids, highest values were shown for **alanine** (MFC = 1.42, P < 0.001), classified as late marker and strong predictor. |
|  | 8032936 | The effects of **acetyl-L-carnitine** (ALCAR) treatment on brain energy state recovery and lactic acid levels […] and a corresponding increase in lactic acid, inorganic phosphate (Pi), AMP, creatine, glycerol 3-phosphate and **alanine** levels. |
|  | 22709675 | ALCAR (**Acetyl-l-carnitine**) attenuated the PTZ induced reduction in [3-(13)C] **alanine** and the increase in dopamine in the HF |
| Alanine AND Carnitine | 26600713 | T2DM (type 2 diabetes mellitus) rats had higher concentrations of α- and β-glucose, but lower concentrations of isoleucine, leucine, valine, glutamine, glycoprotein, lactate, tyrosine, creatine, **alanine**, **carnitine**, and phenylalanine. |
|  | 26595282 | For heart, DOX (Doxorubicin) exposure caused decline of lipid, lactate, leucine, **alanine**, glutamate, choline, xanthine, glycerin, **carnitine**, and fumarate, together with elevation of glutamine, creatine, inosine, taurine and malate. |
|  | 26050356 | The main metabolites contributing to these discriminations, as highlighted by multivariate analysis and confirmed by spectral integration, were formate, tyrosine, β-glucose, inositol, glycine, **carnitine**, glutamine, acetate, **alanine**, valine, isoleucine, |
| Beta.alanine AND TNFa | 24558439 | Early shifts in hepatic oxidative stress and plasma GSH loss preceded a statistically significant rise in **TNF-α**. […] GSSG (oxidized GSH) and **β-alanine**, were positively correlated, and plasma GSH cysteinylglycine, and branched chain amino acids were inversely correlated with hepatic injury. |
| Beta.alanine AND IL.1b | - | - |
| Beta.alanine AND GM.CSF | - | - |
| Beta.alanine AND Carnitine | 25549626 | The effects of dietary **BA (beta alanine)** on selected whole blood **carnitine** esters and their ratios were also not significant. |
|  | 21772982 | (1)H NMR-based metabonomics was used to investigate the multimodal response of mice to malarial parasite infection by P. berghei ANKA. […] Metabolites like kynurenic acid, alanine, **carnitine**, and **β-alanine** showed significant alteration in the liver, |
|  | 2747840 | The uptake of acetyl-L-carnitine was not strictly substrate-specific; gamma-butyrobetaine, L-**carnitine**, L-DABA, and GABA were potent inhibitors, hypotaurine and L-glutamate were moderate inhibitors, and glycine and **beta-alanine** were only weakly inhibitory. |
| Sarcosine AND Carnitine | 21278054 | to identify metabolomic changes induced in the liver by increasing doses of propiconazole in mice […] Many alterations in the levels of biochemicals were found in the glycogen metabolism, glycolysis, lipolysis, **carnitine**, and the tricarboxylic acid cycle pathways […] increases in glycolysis and increases in the levels of spermidine, **sarcosine**, and pseudouridine. |
|  | 26846427 | the inhibited fatty acid oxidation led to a 30-fold decrease in plasma **carnitine** and 9.3-fold decrease in acetylcarnitine at the highest dose of TTP […] TTP administration was associated with […] 1.4-1.6 fold increase in the one-carbon metabolites betaine, dimethylglycine, **sarcosine** and glycine |
|  | 26503852 | **Sarcosine** is found in its free form and is also an intermediate in the catabolic pathways of glycine betaine, **carnitine**, creatine, and glyphosate. |
| Lysine AND Deoxycarnitine (Butyrobetaine) | 23826175 | Administration of TTA led to increased plasma levels of the majority of amino acids, except arginine and **lysine**, which were reduced. […] On the other hand, TTA and fish oil additively reduced plasma levels of the L-carnitine precursor γ-**butyrobetaine** |
|  | 18492302 | Pigs fed the high-**lysine** diet moreover had an increased concentration of trimethyllysine (TML), a reduced mRNA abundance of TML dioxygenase and reduced concentrations of gamma-**butyrobetaine** (BB) in muscle |
| Putrescine AND TNFa | 23327914 | The serum contents of **TNF-α**, IL-1, and IL-6 were increased in NTH, **putrescine**, and cadaverine groups in different degrees at most post injection time points. |
|  | 12860970 | The decrease in **putrescine** levels largely prevented the ability of LPS to trigger **tumor necrosis factor alpha** and TLR2 gene transcription in the mouse brain |
|  | 15909119 | **Putrescine** and overexpression of ODC had similar effects as ROS scavengers in decreasing intracellular ROS […] However, DFMO enhanced the accumulation of ROS, disruption of Delta psi(m) and apoptosis when cells were treated with **TNF-alpha** |
| Enterococcus AND Glutamine | 26566933 | In this study, we characterized genomic diversity in the identified **Enterococcus** hirae ecotypes in order to define further the nature and degree of genome content differences between taxa resolved by cpn60 UT sequences. Genome sequences for six representative isolates (two from each of three ecotypes) were compared. Differences in phosphotransferase systems and amino acid metabolism pathways for **glutamine**, proline and selenocysteine were observed. |
|  | 25493082 | Although not significant, less gut bacterial translocation with **Enterococcus** species developed in the GFO (**glutamine**, fiber, oligosaccharides) group (p = 0.0728) than in the non-GFO group. |
| Enterococcus AND Isoleucine | 23279366 | **E. faecalis** B9510 in a chemically defined medium were determined by single omission experiments. It was observed that eight amino acids (arginine, glycine, histidine, **isoleucine**, leucine, methionine, tryptophan and valine), three B vitamins (nicotinic acid, Ca-pantothenic acid and pyridoxal) and magnesium sulphate were essential for growth. |
|  | 14219061 | Nineteen **enterococcus** isolates, representing all that met the Sherman criteria, required arginine, glutamic acid, histidine, **isoleucine**, leucine, methionine, tryptophan, and valine; |
| Bacteroides AND IL.2 | 16954804 | The increased expression of these genes in **B. vulgatus** colonized mice might be associated with prevention of E. coli mpk triggered colitis in E. coli mpkM/B. vulgatus **IL-2**-/- mice. |
|  | 12851881 | In **IL-2**-/- mice**, B. vulgatus** mpk protects against E. coli mpk-triggered colitis by an unknown mechanism. |
|  | 12812631 | The LPS from **Bacteroides** fragilis was inhibitory to the secretion of **IL-2** from PBMCs |
| Bacteroides AND Aspartic.acid | 7356320 | The lowest uptake of ammonia nitrogen (by **Bacteroides** ruminicola strain 9 ) was observed when the medium contained **aspartic acid**, glutamic acid, threonine, or alanine; |
|  | 13713 | Fermentation of L-**aspartate** by a saccharolytic strain of **Bacteroides** melaninogenicus. |
|  | 8425 | Addition of individual amino acids [...] affected growth rates of an asaccharolytic strain and a saccharolytic strain of **Bacteroides** melaninogenicus. L-**Aspartate** or L-asparagine produced maximal growth enhancement for both strains |
| Streptococcus AND Citrulline | 25645553 | **Citrulline** protects **Streptococcus** pyogenes from acid stress using the arginine deiminase pathway and the F1Fo-ATPase. |
|  | 24144727 | **Streptococcus** pyogenes arginine and **citrulline** catabolism promotes infection and modulates innate immunity. |
|  | 12999796 | The arginine dihydrolase system of **Streptococcus** faecalis. I. Identification of **citrulline** as an intermediate. |
| Streptococcus AND Isoleucine | 18469105 | H(2)O(2) produced on peptone by **S. oligofermentans** was mainly derived from seven L-amino acids, i.e., L-aspartic acid, L-tryptophan, L-lysine, L-**isoleucine**, L-arginine, L-asparagine, and L-glutamine, indicating the possible existence of L-amino acid oxidase (LAAO) that can produce H(2)O(2) from L-amino acids. |
|  | 9485590 | We conclude that ppGpp does accumulate when **S. rattus** and **S. pyogenes** are deprived of **isoleucine** by mupirocin addition. |
|  | 8310179 | Apparent lack of repression of AHAS synthesis by the end-products and reduced sensitivity of **S. bovis** growth to analogues of the branched chain amino acids suggested that secretion of **isoleucine**, leucine and valine in the growth medium may be a consequence of the regulatory features of AHAS. |
| Bifidobacterium AND TNFa | 26134988 | **TNF-α** levels were significantly decreased in the first group after receiving **B. breve** for 3 months. |
|  | 10100903 | Thirty three **Bifidobacterium** strains differentially stimulated the production of H2O2 NO, **TNF-alpha**, and IL-6 in a dose-dependent manner in 24-h cultures. |
|  | 7537575 | We studied the mechanisms of **Bifidobacteria** in antitumor activity using a cell wall preparation (WPG) of B. infantis. […] The mRNA expression of several cytokines (IL-1 beta, IL-6, IL-10, IFN-alpha and **TNF-alpha**) was induced in BALB/c mouse peritoneal cells 3 h after an intraperitoneal injection of WPG (3 h WPG-PEC). |
| Bifidobacterium AND IL.5 | 26565083 | 4 different probiotic strains **Bifidobacterium lactis** [...] Each of these strains demonstrated ability to survive the GI-tract and strain specific effects in producing β-galactosidase, strengthening the gut barrier function after immunological-induced stress and inhibiting Th2 cytokines (IL-4, **IL-5** and IL-13 (≥50%),) |
|  | 25609654 | The intervention group received one capsule of probiotics containing 10(9) cfu **Bifobacterium** bifidum A218, 10(9) cfu Bifidobacterium catenulatum A302, 10(9) cfu Bifidobacterium longum A101, and 10(9) cfu Lactobacillus plantarum A87 . [...] In patients receiving probiotics , levels of serum TNF-α, **IL-5**, IL-6, and endotoxin significantly decreased after six months of treatment. |
|  | 16079493 | We investigated the effect of oral administration of **Bifidobacterium bifidum** G9-1 (BBG9-1) [...] Productions of interferon (IFN)-gamma, interleukin (IL)-4 and **IL-5** from the splenocytes of mice given BBG9-1 were weaker than those of control mice. |
| Bifidobacterium AND IL.2 | 26651209 | Levels of **IL-2**, IFN-γ, IL-10, IL-12p70, and sIgA markedly increased in the NCU-H, NCU-M, NCU-L, and BB12 (**Bifidobacterium**) groups at 10 days of treatment in comparison with the IM group (p < 0.05, p < 0.01), |
|  | 25400245 | **Bifidobacterium longum** MM-2 (MM-2), [....] Oral administration of MM-2 for 17 consecutive days suppressed inflammation in the lower respiratory tract, and decreased virus titers, cell death, and pro-inflammatory cytokines such as IL-6 and TNF-α in bronchoalveolar lavage fluid. The [anti-influenza virus ] mechanism of MM-2 involves innate immunity through [...] significant increase in pulmonary gene expression of NK cell activators such as IFN-γ, **IL-2**, IL-12 and IL-18. |
|  | 15565182 | Enriching the **Bifidobacterium longum**-En treatment with selenium improves the activity of NK and T cells and stimulates the activity of **IL-2** and TNF-alpha in BALB/c mice. |
| Bifidobacterium AND IL.1b | 26515063 | infection with both **Bifidobacterium** **longum** and **Bacteroides** **fragilis** induced the production of significant amounts of **IL-1β** |
|  | 21967771 | The results of DNA microarray analysis of ileal and colonic samples indicated that BB6378 (**Bifidobacterium** **bifidum** OLB6378) increased the gene expression of interleukin (IL)-1α **and IL-1β** |
|  | 18466393 | The reduced level of **IL-1beta** and neutrophil infiltration observed in mice infected with H. pylori following treatment with Lactobacillus casei L26 and **Bifidobacterium** lactis B94 resulted from a modulation of immune response rather than a decrease of H. pylori colonization. |
| Bifidobacterium AND Glutathione | 26706022 | Probiotic capsule consisted of three viable and freeze-dried strains: Lactobacillus acidophilus (2 × 10(9) CFU/g), Lactobacillus casei (2 × 10(9) CFU/g), and **Bifidobacterium** bifidum (2 × 10(9) CFU/g). [...] Taking probiotics resulted in a significant rise in plasma total **glutathione** levels |
|  | 26635786 | In addition, we hypothesized that cysteine plays a role in the oxidative stress response in **B. bifidum**. The use of **glutathione** as an alternative reduced sulfur compound did not alleviate cysteine auxotrophy of this strain, though it was shown to stimulate expression of the genes involved in cysteine biosynthesis, |
|  | 25524403 | mutated clones of probiotic and starter bacteria including Enterococcus faecium, **Bifidobacterium** animalis ssp. lactis, Lactobacillus casei and Lactococcus lactis ssp. lactis by food-level strain development process and clone selection. All mutant clones possessed increased **glutathione** concentration and glutathione reductase activity. |
| Bifidobacterium AND Alanine | 26565082 | In a previous study we have shown that significantly less children developed eczema after probiotic supplementation (**Bifidobacterium bifidum** W23, **Bifidobacterium animalis** subsp. lactis W52 and Lactococcus lactis W58, Ecologic(®)Panda). Metabolites in faecal samples of these 3-month old children were measured by (1)H-nuclear magnetic resonance to investigate possible gut metabolic alterations. Lower amounts of short-chain fatty acids (SCFAs), succinate, phenylalanine and **alanine** were found in faecal samples of children later developing eczema. |
|  | 21148680 | An analysis of the cell-wall composition of strains HM2-1 and HM2-2(T) revealed the presence of glutamic acid, **alanine** and lysine. The presence of fructose-6-phosphate phosphoketolase shows that isolates HM2-1 and HM2-2(T) are members of the genus **Bifidobacterium**. |
|  | 5053884 | A mucoid variant of **Bifidobacterium bifidum** was converted from its normal curved rod or bifid form to a highly branched form when grown in a chemically defined minimal medium. Branching could be prevented by the addition of a mixture of dl-**alanine**, dl-aspartic acid, l(+)-glutamic acid, and dl-serine, but not when any one of these four amino acids was omitted. |
