# Supplementary figures and images for: Multi-Level Integration of Environmentally Perturbed Internal Phenotypes Reveals Key Points of Connectivity between Them
Source: Front Physiol. 2017 Jun 12;8:388. doi: 10.3389/fphys.2017.00388 (PMC5467433; doi:10.3389/fphys.2017.00388)

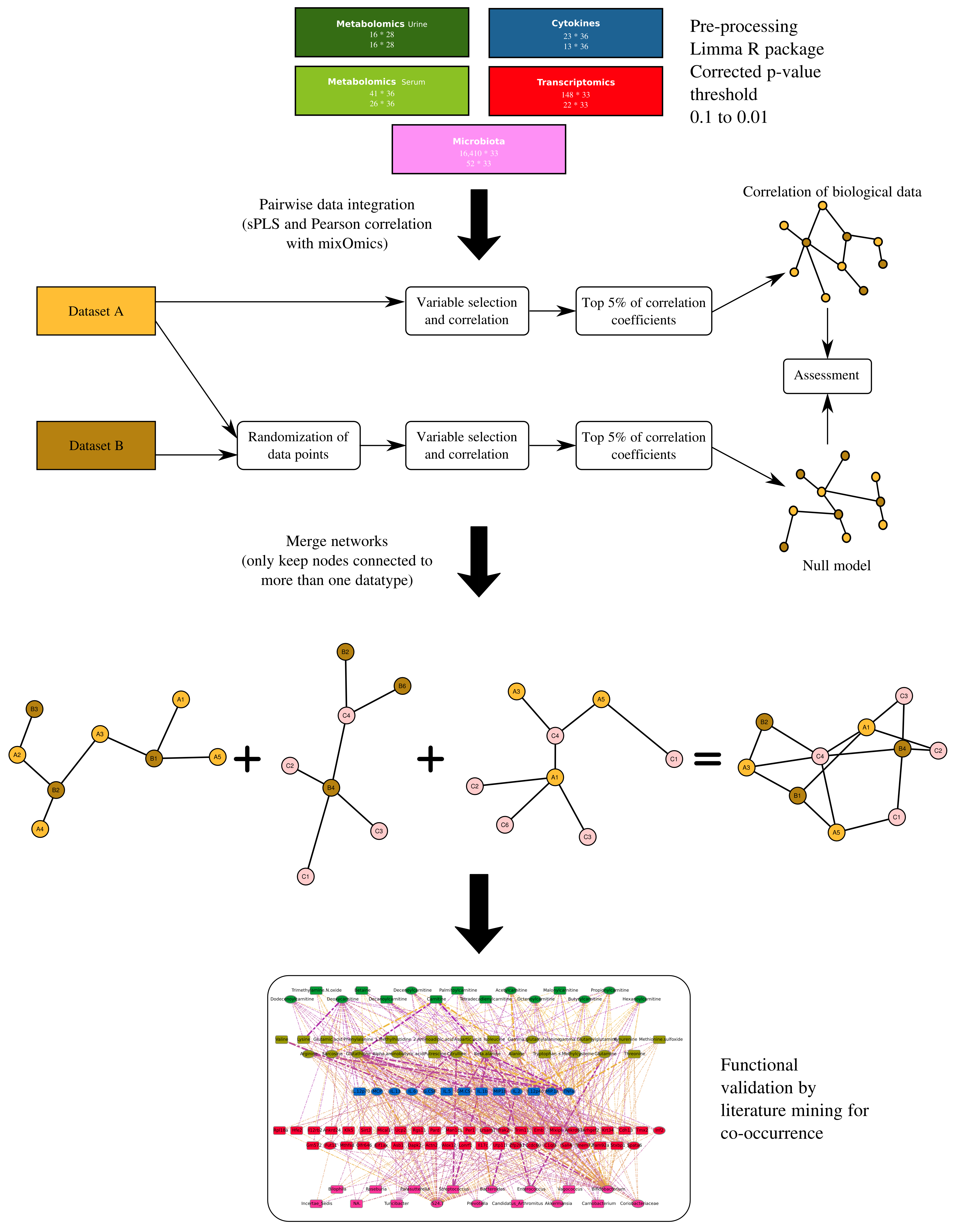

Supplement: Supplementary file 5 [file Image1.PNG]

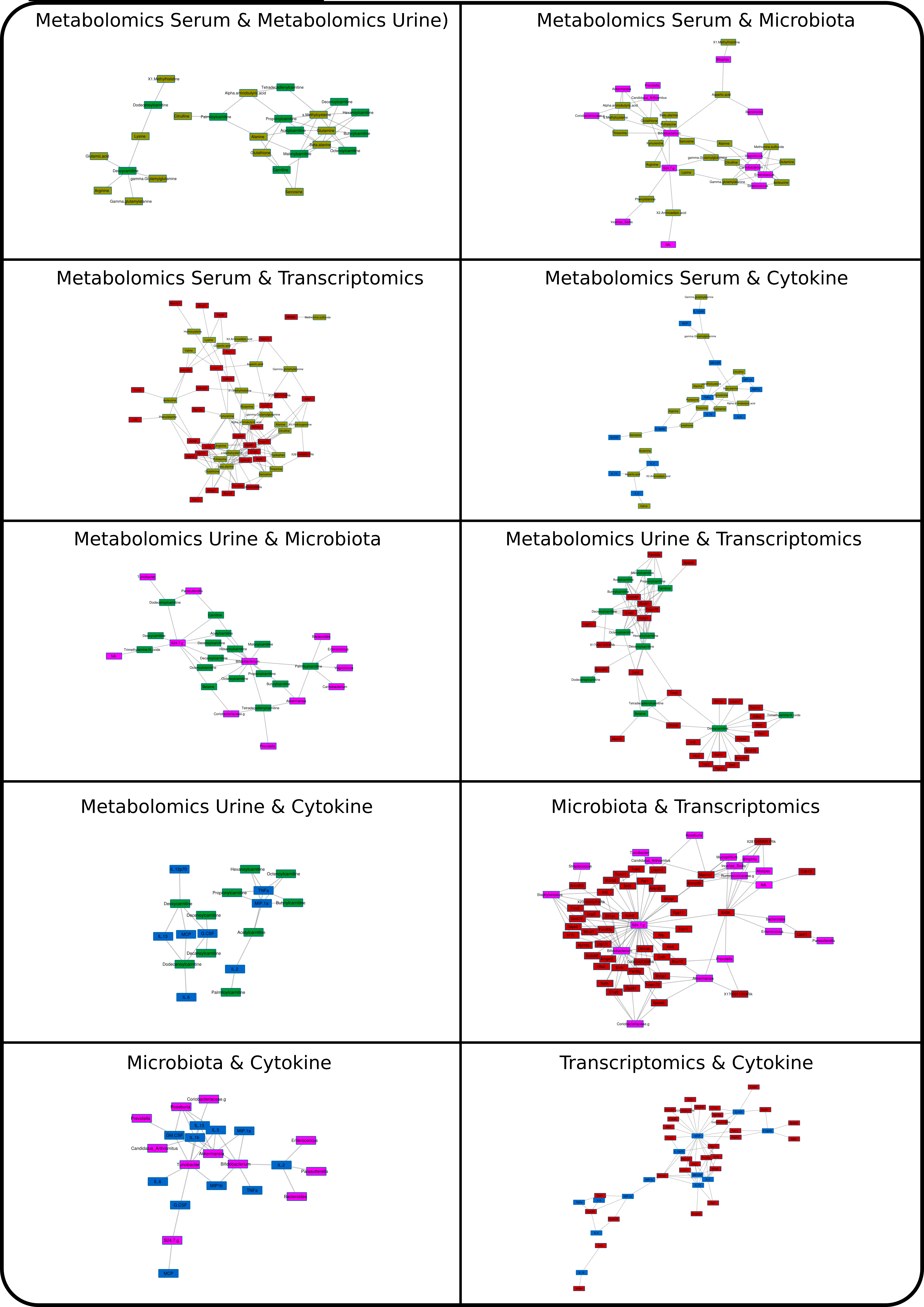

Supplement: Supplementary file 6 [file Image2.PNG]

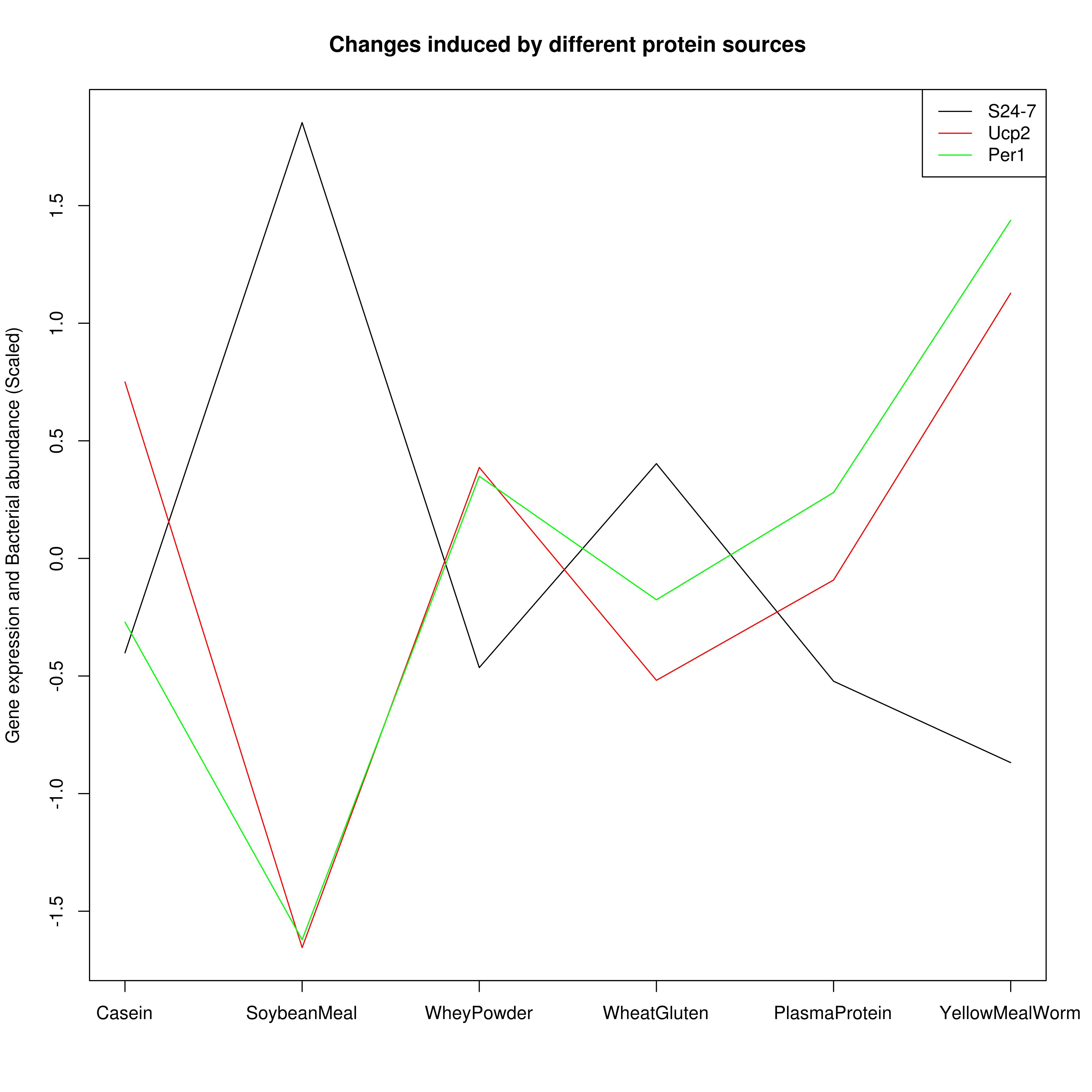

Supplement: Supplementary file 7 [file Image3.png]

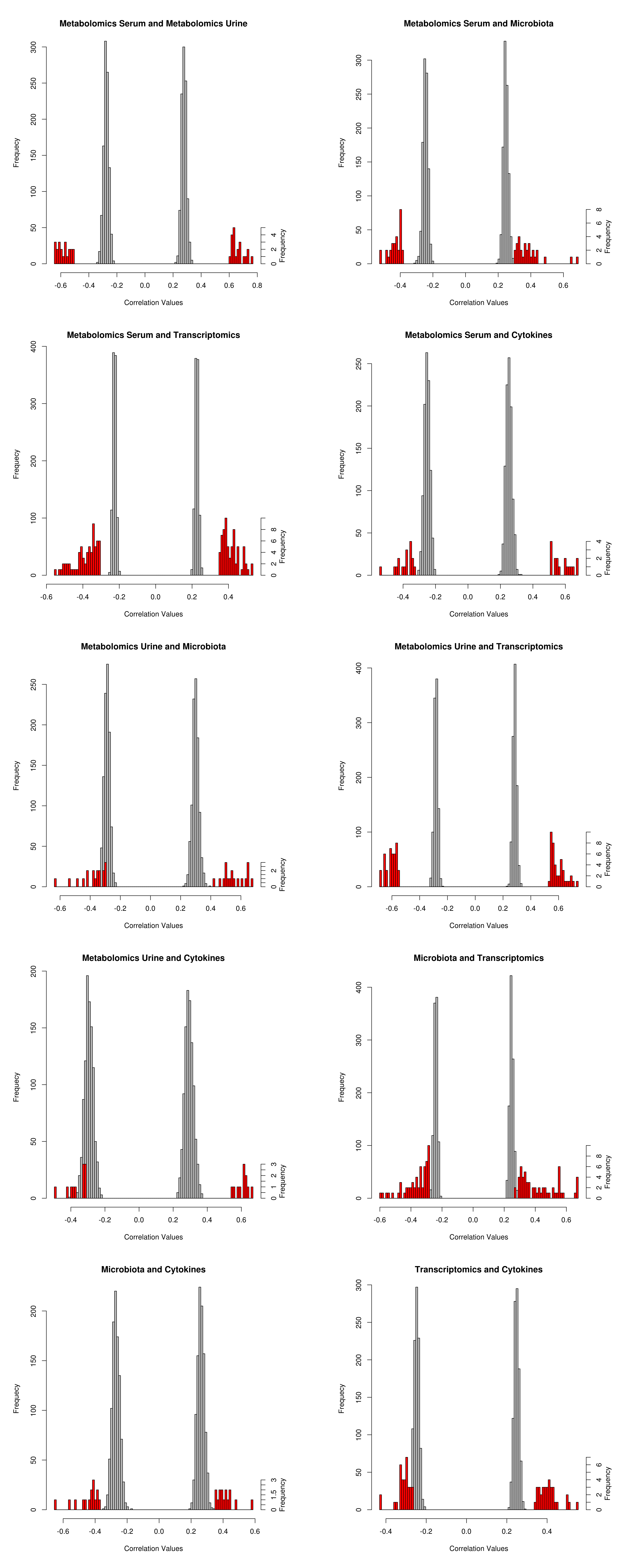

Supplement: Supplementary file 8 [file Image4.png]
